# The psc-CVM assessment system: A three-stage type system for CVM assessment based on deep learning

**DOI:** 10.1186/s12903-023-03266-7

**Published:** 2023-08-12

**Authors:** Hairui Li, Haizhen Li, Lingjun Yuan, Chao Liu, Shengzhao Xiao, Zhen Liu, Guoli Zhou, Ting Dong, Ningjuan Ouyang, Lu Liu, Chenglong Ma, Yang Feng, Youyi Zheng, Lunguo Xia, Bing Fang

**Affiliations:** 1 Department of Orthodontics, Shanghai Ninth People’s Hospital affiliated to Shanghai Jiao Tong University School of Medicine, Shanghai, 200011 China; 2 Chohotech Inc, Hangzhou, China; 3 Translational Medicine Research Platform of Oral Biomechanics and Artificial Intelligence, Department of Orthodontics, Shanghai Ninth People’s Hospital affiliated to Shanghai Jiao Tong University School of Medicine, Shanghai, 200011 China; 4 State Key Lab of CAD&CG, Zhejiang University, Hangzhou, China

**Keywords:** Deep learning, Cervical vertebral maturation (CVM) assessment, Lateral cephalogram

## Abstract

**Background:**

Many scholars have proven cervical vertebral maturation (CVM) method can predict the growth and development and assist in choosing the best time for treatment. However, assessing CVM is a complex process. The experience and seniority of the clinicians have an enormous impact on judgment. This study aims to establish a fully automated, high-accuracy CVM assessment system called the psc-CVM assessment system, based on deep learning, to provide valuable reference information for the growth period determination.

**Methods:**

This study used 10,200 lateral cephalograms as the data set (7111 in train set, 1544 in validation set and 1545 in test set) to train the system. The psc-CVM assessment system is designed as three parts with different roles, each operating in a specific order. 1) Position Network for locating the position of cervical vertebrae; 2) Shape Recognition Network for recognizing and extracting the shapes of cervical vertebrae; and 3) CVM Assessment Network for assessing CVM according to the shapes of cervical vertebrae. Statistical analysis was conducted to detect the performance of the system and the agreement of CVM assessment between the system and the expert panel. Heat maps were analyzed to understand better what the system had learned. The area of the third (C3), fourth (C4) cervical vertebrae and the lower edge of second (C2) cervical vertebrae were activated when the system was assessing the images.

**Results:**

The system has achieved good performance for CVM assessment with an average AUC (the area under the curve) of 0.94 and total accuracy of 70.42%, as evaluated on the test set. The Cohen's Kappa between the system and the expert panel is 0.645. The weighted Kappa between the system and the expert panel is 0.844. The overall ICC between the psc-CVM assessment system and the expert panel was 0.946. The F1 score rank for the psc-CVM assessment system was: CVS (cervical vertebral maturation stage) 6 > CVS1 > CVS4 > CVS5 > CVS3 > CVS2.

**Conclusions:**

The results showed that the psc-CVM assessment system achieved high accuracy in CVM assessment. The system in this study was significantly consistent with expert panels in CVM assessment, indicating that the system can be used as an efficient, accurate, and stable diagnostic aid to provide a clinical aid for determining growth and developmental stages by CVM.

**Supplementary Information:**

The online version contains supplementary material available at 10.1186/s12903-023-03266-7.

## Background

Accurate assessment of growth and development plays an essential role in the work of clinicians. The most variables are dental and craniomaxillofacial growth and development during childhood and adolescence. They are susceptible to the development of oral diseases caused by various factors, such as deformity of tooth maxillofacial [1, 2], the late eruption of teeth due to rickets and nutritional deficiencies [3, 4], abnormal number [5] and shape of teeth [6] due to hereditary diseases. Therefore, clinicians must adequately understand and master the typical growth and development characteristics and their influencing factors to diagnose, prevent, and treat diseases.

Many scholars have proven that cervical vertebral maturation (CVM) can predict the growth and development of the face and assist in choosing the best time for treatment [7–10]. However, assessing the CVM is a complex and time-consuming process. The experience and seniority of the clinicians have an enormous impact on judgment. Gabriel et al. [11] tested the reproducibility of the CVM method with ten orthodontists and showed an interobserver agreement of less than 50%. Nestman et al. [12] also reported significant variability in the results of clinicians applying cervical vertebrae to assess the growth periods. This leads to difficulties in determining the optimal timing of treatment during adolescence and is a pressing clinical problem.

Recent research has shown that artificial intelligence (AI) has vital image target detection and classification capabilities [13, 14]. In medical image analysis, AI has been successfully applied to many projects, significantly improving the recognition accuracy of images. Huang et al. [15] proposed a new convolutional neural network for detecting and classifying interstitial lung disease, achieving an average F1 score (a harmonic mean between recall and precision) of 0.9654. Wu et al. [16] present a deep convolutional neural network for breast cancer screening classification, trained, and evaluated on over 200,000 exams (over 1,000,000 images), achieving an AUC (area under the ROC curve) of 0.895 in predicting the presence of cancer in the breast when tested on the screening population. Nasrullah et al. [17] proposed a system based on deep learning for lung nodule detection and classification. The proposed system was evaluated on LIDC-IDRI datasets, achieving a sensitivity of 94% and a specificity of 91%.

However, the number of studies that have applied AI to CVM assessment could be more significant. Most of these studies used measurements of vertebral body morphology to train AI models, such as the ratio of the posterior height to the anterior height of vertebral bodies [18–22]. This approach is labor-intensive and time-consuming and results in a limited number of lateral cephalograms for training, while the sample size for training dramatically affects the final performance of the AI model. On the other hand, CVM is commonly evaluated in clinical settings by direct vision, allowing easy access to many samples for training. In addition, currently, deep learning algorithms are the dominant technology in the field of artificial intelligence. It can perform feature extraction in an automated manner, which allows researchers to extract differentiated features with minimal domain knowledge and human effort. Thus, combining the two makes it possible to train highly performing-AI models.

This study aims to establish a CVM assessment system called the psc-CVM assessment system based on deep learning with 10,200 lateral cephalograms to provide valuable reference information for clinicians in diagnosis and treatment planning and help clinicians in underdeveloped areas or inexperienced to make a stable and reliable treatment decision.

## Methods

### Ethical approval

This study was approved by the Ethics Committee of Shanghai Ninth People's Hospital. Lateral cephalograms for research were collected from patients screened or treated for malformations at Shanghai Ninth People's Hospital. Obtained Written/verbal informed consent from all participating patients, and all participating patients had the right to withdraw from this study at any time. This study was approved by the Institutional Review Board (IRB: SH9H-2020-TK400-1) and was conducted by the Declaration of Helsinki and Ethics and governance of artificial intelligence for health under WHO guidance.

### Data set creation and distribution

The subjects of this study were consecutive cases, and 15,000 lateral cephalograms were collected from patients admitted to the Department of Orthodontics of Shanghai Ninth People's Hospital from 2015 to 2022. Subjects without congenital or acquired malformation of the cervical vertebrae, trauma and/or operation in the head and neck region, any disorder that could interfere with bone development, any systemic disease and/or growth and development retardation, and any congenital and/or acquired malformations in the head and neck region were included. All lateral cephalograms have at least clear visualization of second (C2), third (C3), and fourth (C4) cervical vertebrae. All the images were stored and read in JPG format.

The stages of cervical vertebral maturation in the modified version of the method presented here are illustrated diagrammatically in Table 1 [23]. The six stages are defined as follows:

- Cervical stage 1 (CVS1). The lower borders of all the three vertebrae (C2-C4) are flat. The bodies of both C3 and C4 are trapezoid in shape (the superior border of the vertebral body is tapered from posterior to anterior). The peak in mandibular growth will occur on average 2 years after this stage.
- Cervical stage 2 (CVS2). A concavity is present at the lower border of C2 (in four of five cases, with the remaining subjects still showing a cervical stage 1). The bodies of both C3 and C4 are still trapezoid in shape. The peak in mandibular growth will occur on average 1 year after this stage.
- Cervical stage 3 (CVS3). Concavities at the lower borders of both C2 and C3 are present. The bodies of C3 and C4 may be either trapezoid or rectangular horizontal in shape. The peak in mandibular growth will occur during the year after this stage.
- Cervical stage 4 (CVS4). Concavities at the lower borders of C2, C3, and C4 now are present. The bodies of both C3 and C4 are rectangular horizontal in shape. The peak in mandibular growth has occurred within 1 or 2 years before this stage.
- Cervical stage 5 (CVS5). The concavities at the lower borders of C2, C3, and C4 still are present. At least one of the bodies of C3 and C4 is squared in shape. If not squared, the body of the other cervical vertebra still is rectangular horizontal. The peak in mandibular growth has ended at least 1 year before this stage.
- Cervical stage 6 (CVS6). The concavities at the lower borders of C2, C3, and C4 still are evident. At least one of the bodies of C3 and C4 is rectangular vertical in shape. If not rectangular vertical, the body of the other cervical vertebra is squared. The peak in mandibular growth has ended at least 2 years before this stage.

**Table 1:**
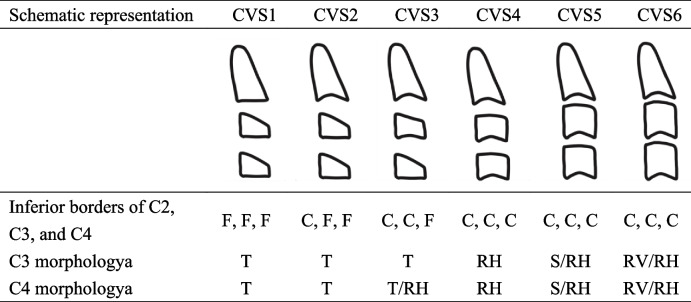
The six stages of cervical vertebral maturation

*F* Flat, *C* Concavity, *T* Trapezoid, *R* Rectangular Horizontal, *S* Square, *RV* Rectangular Vertical

The cervical vertebral maturation stage (CVS) of patients was determined as described by Baccetti et al. [23]. The CVS of each lateral cephalogram was determined by two independent orthodontists with more than ten years of experience. If no agreement could be reached, a senior orthodontist who had been treating malocclusion for more than 25 years was consulted.

**Fig. 1:**
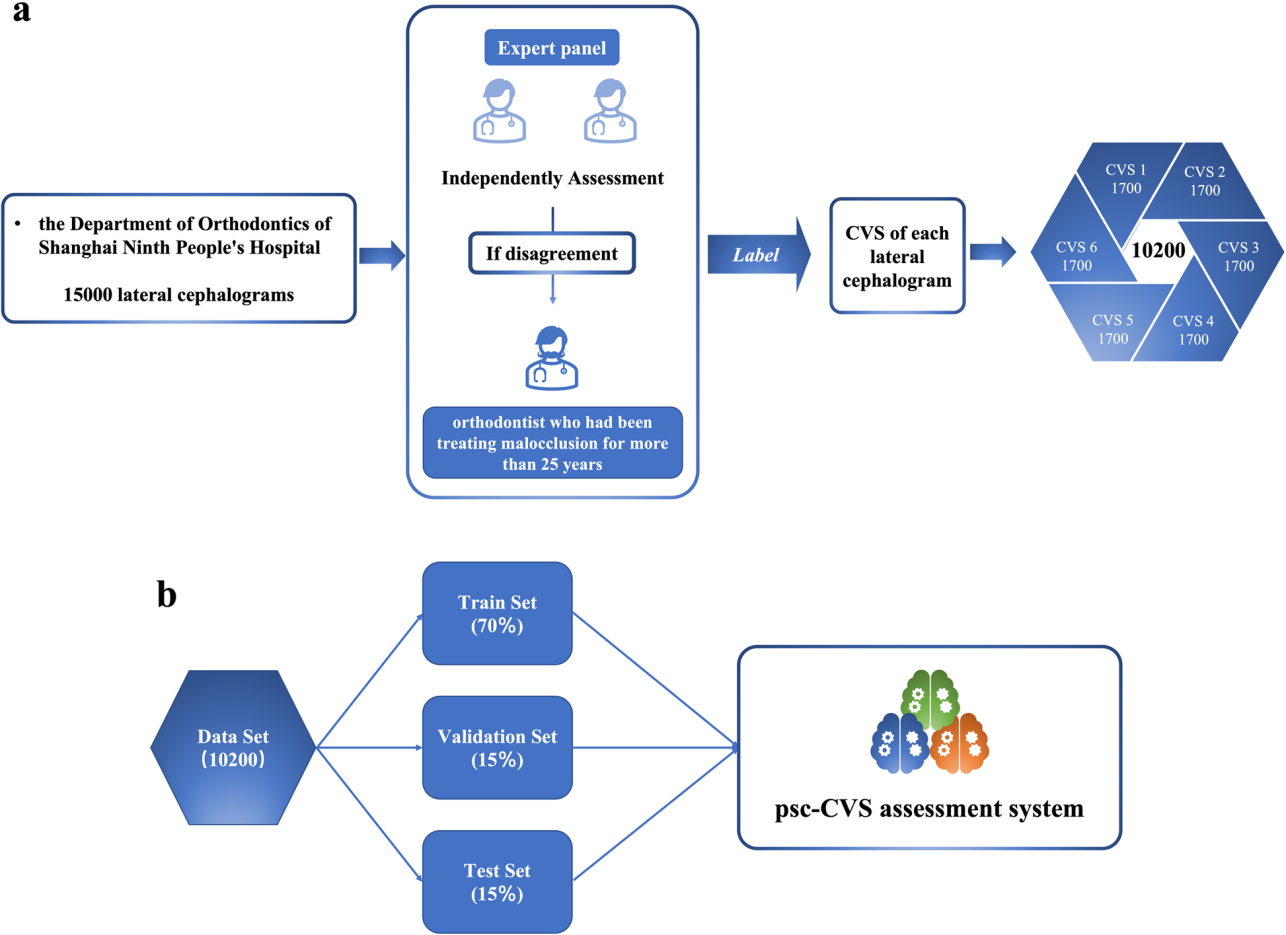
The entire process of Data set creation and distribution. **a** A total of 15,000 lateral cephalograms were collected from patients admitted to the Department of Orthodontics of Shanghai Ninth People's Hospital. The CVS of each lateral cephalogram was determined by expert panel. Finally, a data set with 10,200 samples was established, and each CVS had 1700 samples. **b** 70% of the data set is used as the training set, 15% as the validation set, and 15% as the test set, and they are independent of each other without overlap

In order to balance the sample size of each CVS, the redundant lateral cephalograms were removed. Finally, we established a data set with 10,200 samples; each CVS had 1700 samples. 70% are used as the training set for training the CVM assessment system called the psc-CVM assessment system, 15% as the validation set for tuning the hyperparameters of the system, and 15% as the test set for evaluating the performance of the system, and they are independent of each other without overlap. The entire process in this part is briefly presented in Fig. 1.

### Working framework and training details of the psc-CVM assessment system

**Fig. 2:**
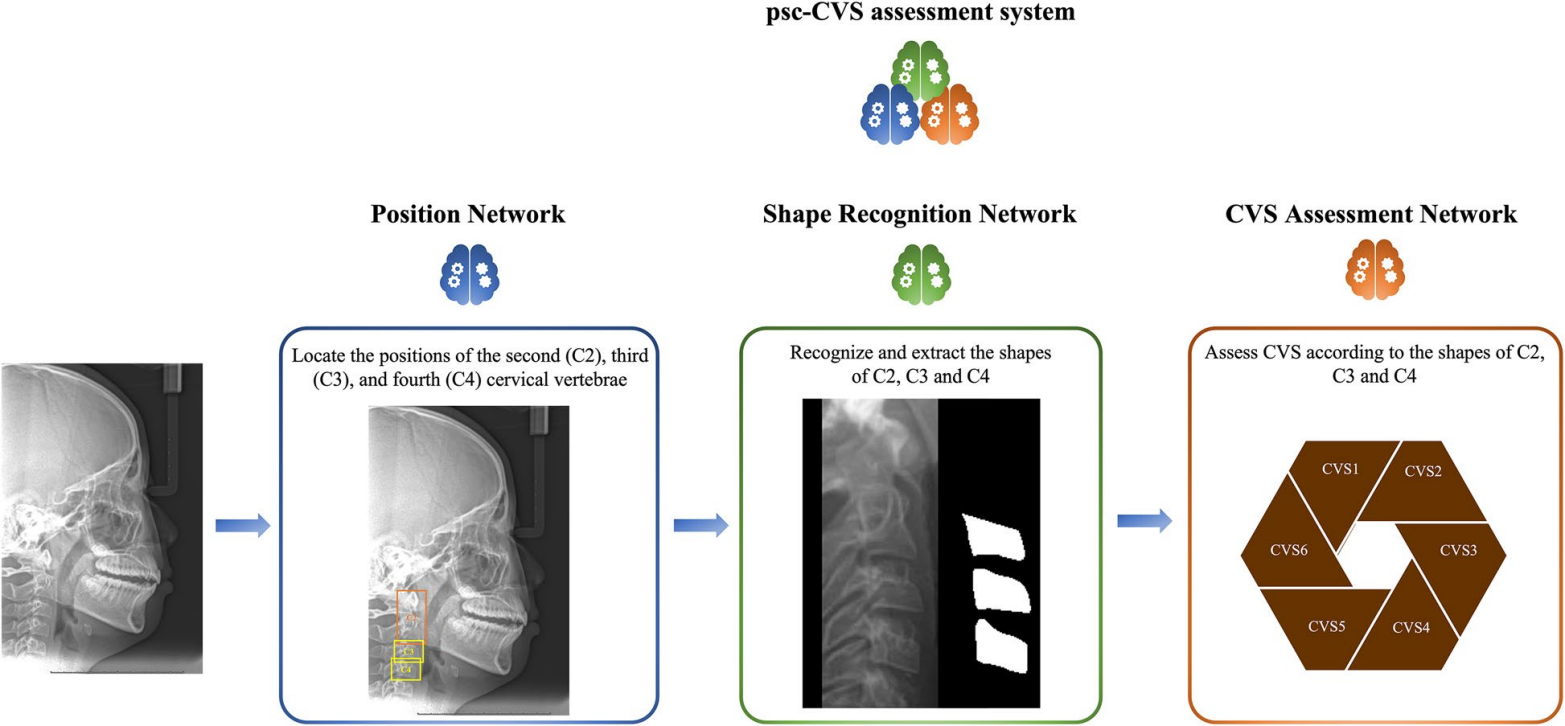
The working framework of psc-CVM assessment system. The working framework is designed as three parts with different roles, each operating in a specific order. Position Network is intended to locate the position of the second (C2), third (C3), and fourth (C4) cervical vertebrae. Shape Recognition Network is intended to recognize and extract C2, C3, and C4 shapes. CVM Assessment Network is intended to assess CVS according to the shapes of C2, C3 and C4

Due to the similarity between different vertebrae of the same lateral cephalogram, and the differences in vertebrae morphology in other CVS, we combine the lateral cephalogram features and the advantages of various networks to build a working framework with both accuracy and speed for the psc-CVM assessment system. Specifically, the working framework is designed as three parts with different roles, each operating in a specific order. The working framework is shown in Fig. 2. Our training was performed on the server of the computing platform with the NVIDIA GTX 3080 graphic processing unit. More training details is illustrated in an additional file [see Additional file 1].

1) Position Network for locating the position of the second (C2), third (C3), and fourth (C4) cervical vertebrae. YOLOv3 [24] was selected as the core of operation for this phase. 2) Shape Recognition Network for recognizing and extracting C2, C3, and C4 shapes. This study proposed the method of the accurate extraction of vertebral contours by predicting dense key points using the relation between contour key points and morphology, modeling shape recognition as a heatmap regression problem. Select 1000 lateral cephalograms from the training set and build a data set for this stage of training. Inclusion criteria: In lateral cephalograms, the data set will be included as long as there is a bony protrusion on the lower border of any one of C2, C3, and C4 (no distinction between front and back borders). The bony protrusions are bony structures that may exist in the lower borders of C2, C3, C4, which are not connected to the vertebral body but are present in isolated areas of the bone. These bony structures may make the assessment difficult because they can be mistaken for extensions of the lower bound [25]. We randomly selected a doctor from the expert panel, and the doctor marked the actual contour of the cervical spine. The final sample was a doctor who had been in orthodontic treatment for over ten years. The data obtained is used for the network training at this stage. 3) CVM Assessment Network for assessing CVS according to C2, C3, and C4 shapes. We combined the results of the first two steps. The images in the C2-C4 range were cropped according to the prediction rectangle in the first step. The corresponding vertebral binary masks were generated based on the output of the second step, which was concatenated and used as inputs. This phase built the classification model, using ConvNext [26] as the backbone. Used soft-label [27] as the prediction target of the network.

### Evaluation metrics for training results

From the deep learning perspective, cervical bone age staging is a typical classification task. Therefore, the evaluation index of the classification task is used to judge whether the system is well-trained. The evaluation indicators of classification tasks mainly include the loss-accuracy rate curve, confusion matrix, accuracy rate, precision rate, recall rate, F1 score (F1-Score), ROC curve, and accuracy rate-recall rate curve.

#### Loss-accuracy curve (Loss-accuracy curve)

It can reflect whether the system has an underfitting or overfitting phenomenon, and these two phenomena will not appear in a well-trained system.

#### Confusion matrix

It can intuitively reflect the accuracy and deviation of the system classification. The more concentrated the sample distribution on the diagonal, the better the training result of the system.

#### Accuracy, precision, recall

Accuracy reflects the overall classification accuracy of the system. The precision rate measures how many of the samples predicted by the system as positive classes are true positive classes, and the recall rate reflects the degree of coverage of the system for the actual positive class samples. The higher the value of the three, the better the training result of the system.

#### F1 score (F1 score)

F1 score is a comprehensive evaluation index of precision and recall, with a maximum value of 1 and a minimum value of 0. The closer the F1 score is to 1, the better the training result of the system.

#### ROC curve and AUC value

The ROC curve can reflect the comprehensive ability of the system to identify positive and negative examples. The area under the ROC curve is the AUC (Area Under Curve) value. The larger the AUC area, the better the classification ability of the system (the better the training result).

#### Precision-recall curve

Focuses on the accuracy of the system's recognition of positive examples. AP (Average Precision) value is the area under the PR curve; the more significant the AP value, the better the training result of the system.

#### Statistical analysis and evaluation

All the statistical analysis were performed using SPSS Statistics Version 22.0. Used the statistics of Kappa (Cohen’s, Weighted) and intraclass correlation coefficients (ICCs) to evaluate the agreement of CVM assessment between the system and the expert panel.

## Results

**Fig. 3:**
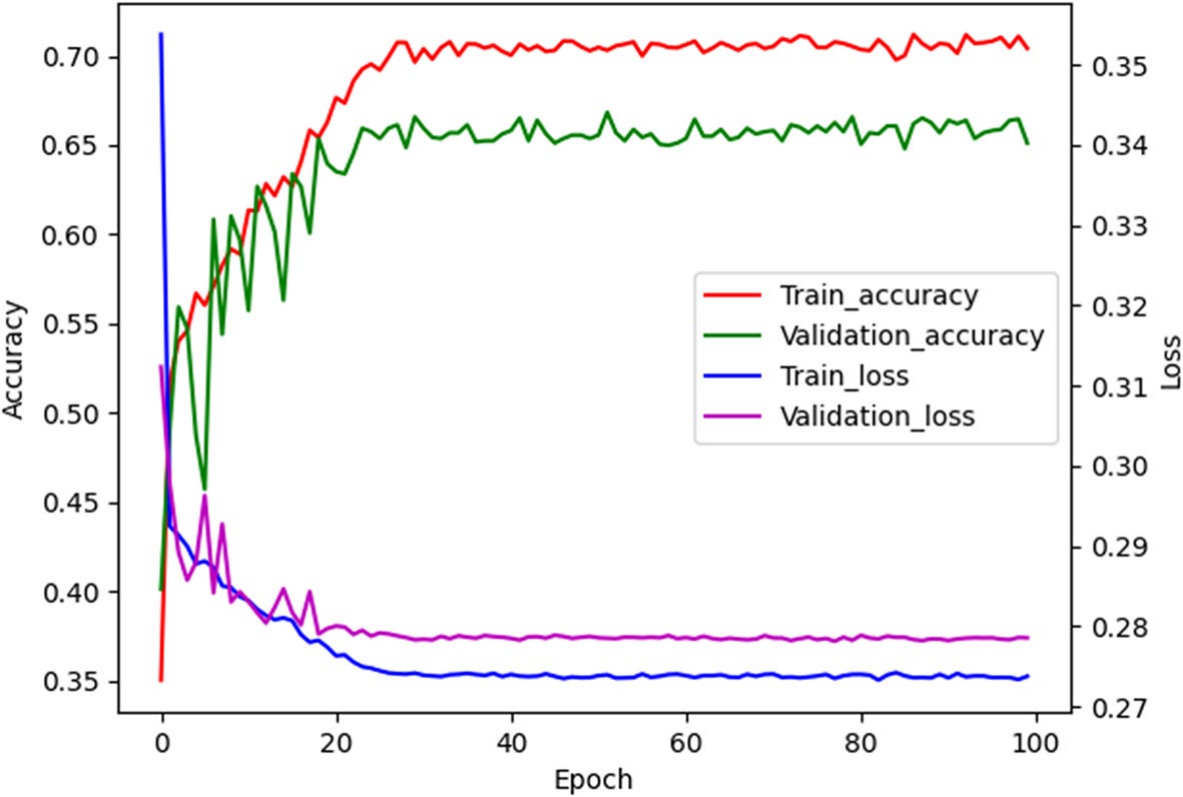
Loss-Accuracy curve for the psc-CVM assessment system. The horizontal axis of the graph represents the training epochs. The left vertical axis of the graph represents the classification accuracy, and the right vertical axis of the graph represents the loss value. The loss function for CVM assessment decreases significantly at the beginning of training and converges well in the first ten epochs. As the loss function decreases, the system's classification accuracy increases rapidly

The Loss-Accuracy curve of the system are shown in Fig. 3. The loss function decreases significantly at the beginning of training and converges well in the first 20 epochs. After 100 epochs of training, it can be observed that the loss values of the training set and the validation set are relatively close, indicating that the system did not experience overfitting. Furthermore, the system's classification accuracy increases rapidly as the loss function decreases. The classification accuracy of the system on both the training and validation sets is very similar, suggesting the absence of underfitting.

To visualize the performance of the psc-CVM assessment system, we plotted the confusion matrix of the CVM assessment results of the system on the test set. The confusion matrix shows that the samples are mainly concentrated around the diagonal line, and a few are away from the diagonal line. The CVM assessment results of the system are always in the correct category. Even if there are errors, the system always tends to assign them to a similar category rather than one far from the correct one.

**Fig. 4:**
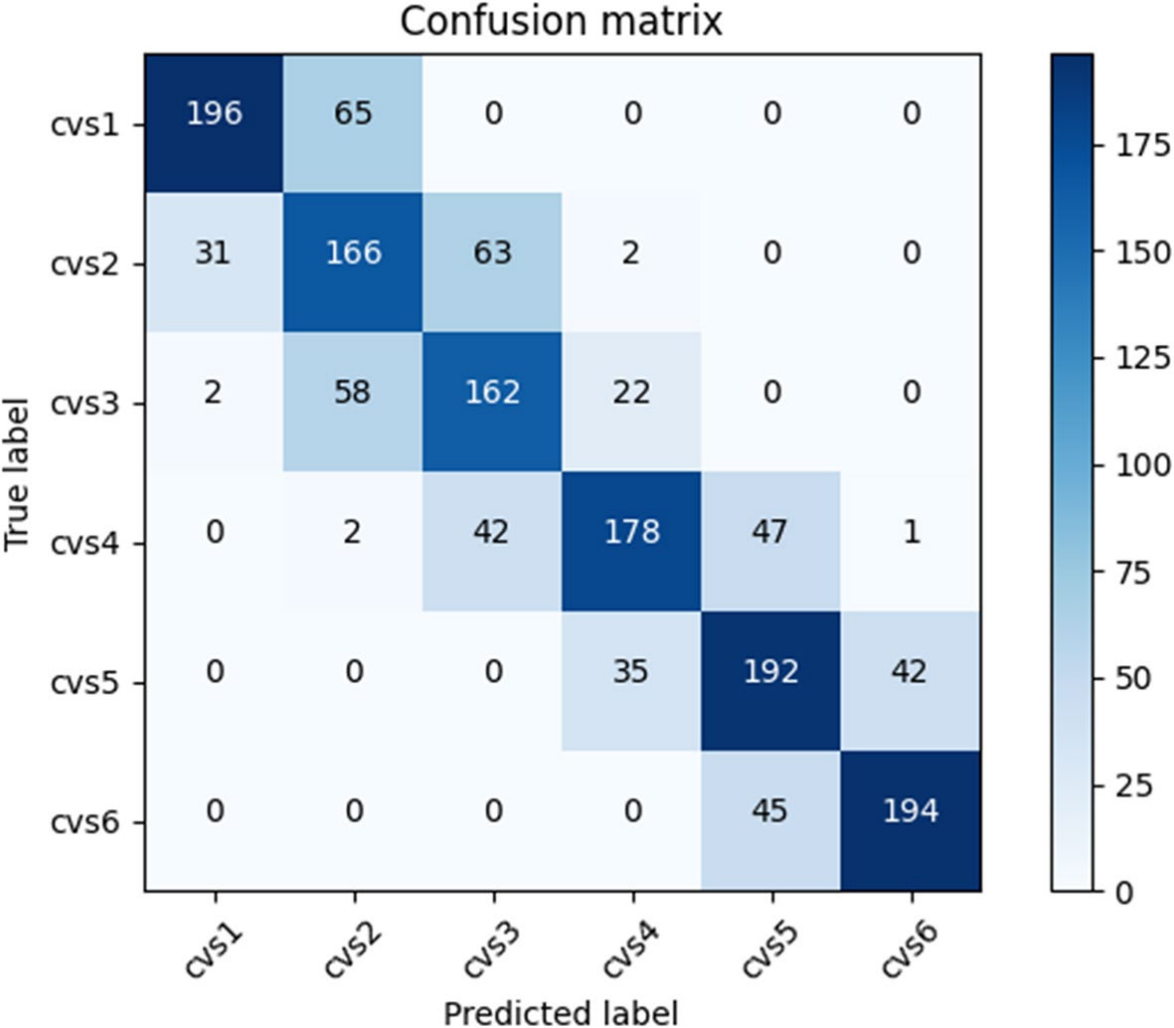
Confusion matrix obtained using the psc-CVM assessment system on test set

In order to further describe the classification ability of the system for each CVS, we calculated the accuracy, recall, and F1 values for each subclass based on the confusion matrix (Fig. 4), and the results are shown in Table 2.

**Table 2:** Precision, recall rates and F1 score of the psc-CVM assessment system on test set for each CVM subgroup

	**Precision**	**Recall**	**F1 score**	**Sample size of each subgroup**
CVS 1	0.8559	0.7509	0.8000	261
CVS 2	0.5704	0.6335	0.6003	262
CVS 3	0.6067	0.6639	0.6340	244
CVS 4	0.7510	0.6592	0.7021	270
CVS 5	0.6760	0.7137	0.6943	269
CVS 6	0.8185	0.8117	0.8151	239

Precision = TP/TP + FP; Recall = TP/TP + FN; F1 score = 2*Precision*Recall/Precision + Recall. TP is true CVS, cervical vertebral maturation stage; FP is false positive, FN is false negative, and TN is true negative. Precision, Recall and F1 score are calculated with confusion matrix in Fig. 4

The Cohen's Kappa between the system and the expert panel is 0.645, indicating a substantial agreement. The weighted Kappa between the system and the expert panel is 0.844, indicating an almost perfect agreement. Kappa < 0.2 indicates that the degree of consistency is slight; between 0.2 and 0.4, the degree of consistency is fair; between 0.4 and 0.6, the degree of consistency is moderate; between 0.6 and 0.8, the degree of consistency is substantial; between 0.8 and 1.0 between shows an almost perfect degree of consistency.

The overall ICC between the psc-CVM assessment system and the expert panel was 0.946. The accuracy of the system in the test set is 70.42%. Comparing the F1 values of the six periods of CVS, we can conclude that the discrimination ability of the system for each subclass is from highest to lowest: CVS6 > CVS1 > CVS4 > CVS5 > CVS3 > CVS2, and the system has poor discrimination ability for CVS2 and CVS3, but better discrimination ability for CS1 and CS6.

**Fig. 5:**
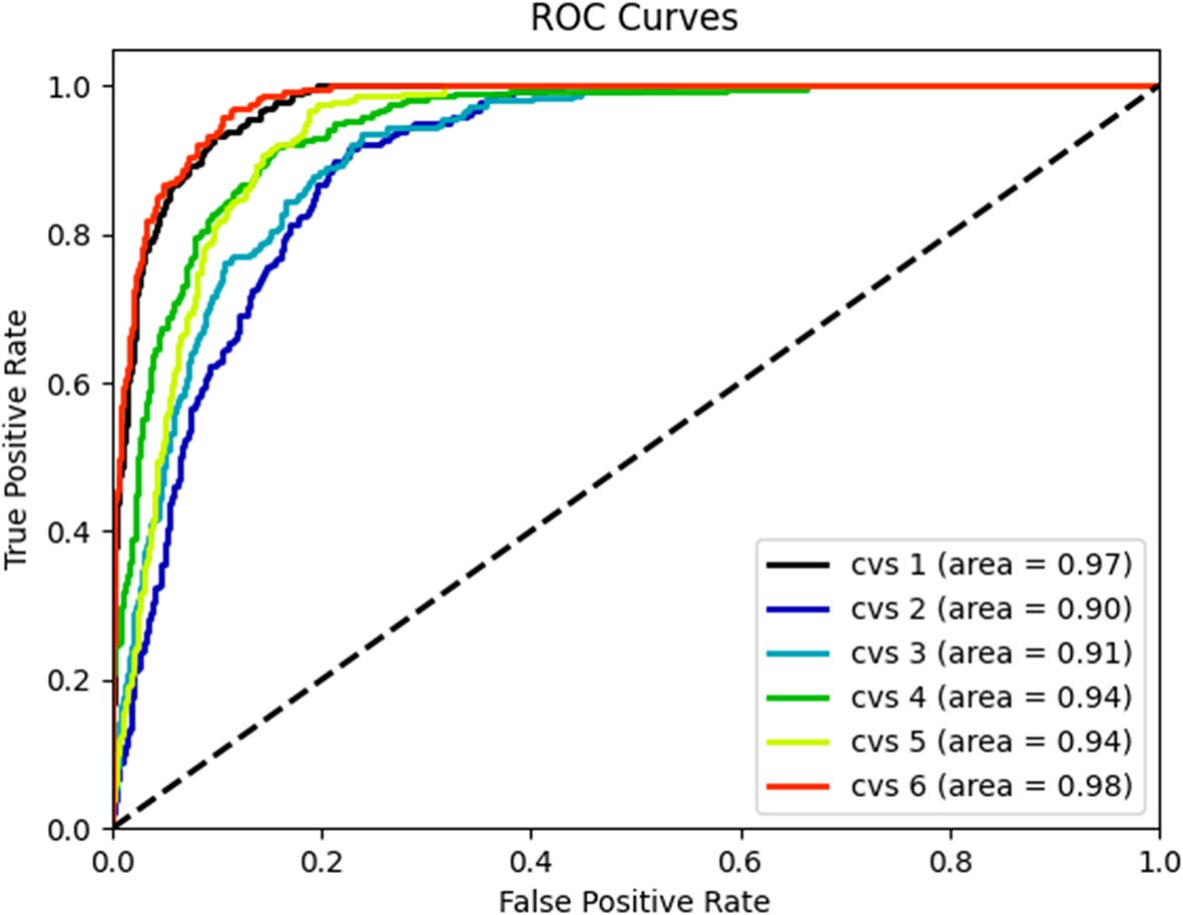
ROC curves of the psc-CVM assessment system for the CVM assessment. ROC, receiver operating characteristic

The ROC curves of the classification results on the test set of the psc-CVM assessment system are shown in Fig. 5. The ROC curves show that the AUC values of the psc-CVM assessment system in this study are above 0.90 on the test set. The average AUC is 0.94. However, the ROC curves for the multiclassification problem are more insensitive to the class imbalance problem. The reason may be related to the method of plotting ROC curves for multiclassification problems, which need to be transformed into multiple binary classification problems when dealing with multiclassification problems. This method leads to a severe sample imbalance (the ratio of positive to negative samples in this study is about 1:5), so the ROC values of each subclass are very high, and the ROC curves of the multiclassification problem are different from the actual classifier performance.

**Fig. 6:**
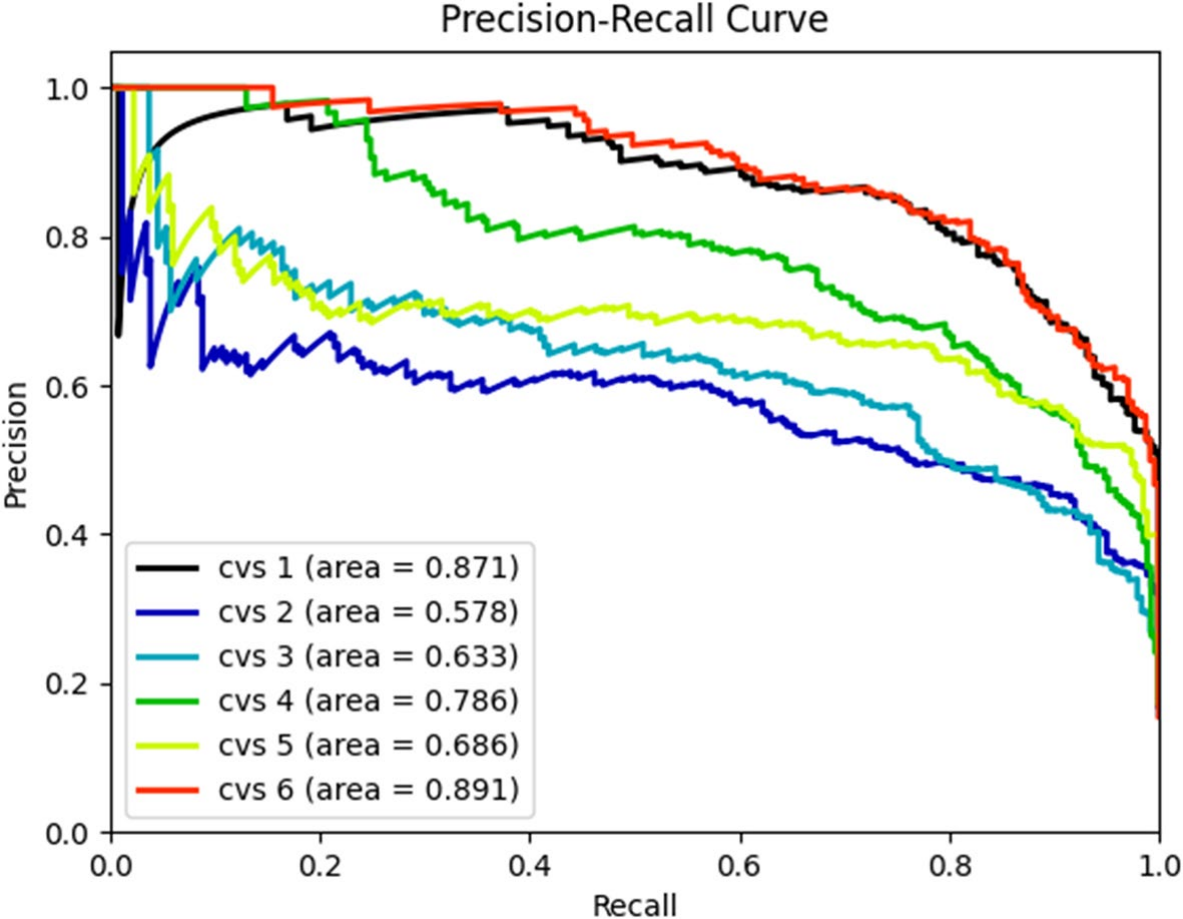
P-R curves of the psc-CVM assessment system for the CVM assessment. P-R, precision-recall. x Heatmaps illustrating which parts of the lateral cephalogram contributed to the prediction results. The activation regions of the system were in the lower edge of C3, C4, and the lower edge of C2

On the other hand, the P-R curve is more focused on positive samples and has better sensitivity in dealing with the multi-classification problem. From Fig. 6, we can find that the system has the best diagnosis for CS1 and CS6 with AP values of 0.871 and 0.891, respectively, and the worst diagnosis for CS2 and CS3 phases with AP values of 0.578 and 0.633, respectively.

**Fig. 7:**
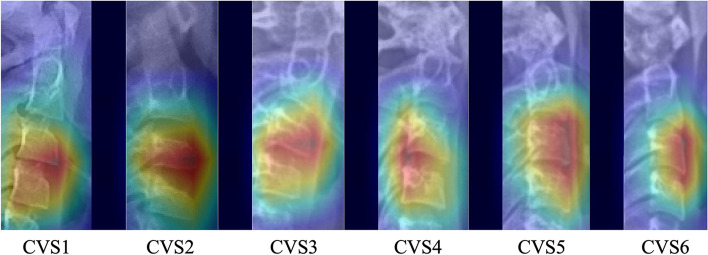
Heatmaps illustrating which parts of the lateral cephalogram contributed to the prediction results. The activation regions of the system were in the lower edge of C3, C4, and the lower edge of C2

A heat map was generated using class activation mapping (CAM). The method activates the significant regions influencing the diagnostic outcome during inference. In this study, the activation regions of the system were in the lower edge of C3, C4, and the lower edge of C2 (Fig. 7). This is an indication of a well-trained system that effectively uses the information in the lateral cephalogram. Clinicians also pay attention to information such as vertebrae contour and vertebrae spacing in assessing CVM, which indicates an agreement between the system and clinicians' experience in predicting bone age.

The system's speed was approximately 26.51 ms for analyzing a single lateral cephalogram.

## Discussion

In clinical practice, it is crucial to determine the growth stage accurately. Orthodontists, pediatric dentists, and even pediatricians often use bone age to determine the growth stage to guide treatment planning and drug selection, especially in the treatment of growth modification and intervention before the growth spurt. Using growth and development potential to treat disorders can lead to better efficacy.

The CVM described by Baccetti et al. [23] is widely used in clinical practice, but the experience and seniority of the clinicians have an enormous impact on judgment. According to certain scholars, the CVM may be considered subjective, leading to potentially questionable outcomes [11, 12].

Applying artificial intelligence to evaluation may be a solution to the problem. Kök et al. [28] defined 19 reference points on C2, C3 and C4 and performed 20 different linear measurements to create a dataset to train neural network models. They compared the accuracy of several models, showing that the model with the highest accuracy, ANN-6, had an accuracy of 0.8687. The AI model and human observers observed an average of 58.3% agreement. Amasya et al. [19] developed five different machine learning classifier models and compared their performance for cervical vertebral maturation (CVM) analysis. Among the CVM stage classifier models, the best result was achieved using the ANN model (κ = 0.926). Amasya et al. [18] then marked 26 reference points on the vertebrae of the lateral cephalograms for measurement and built a dataset to compare the model's output with the results of human observers to validate the effectiveness of the CVS AI model.

Most of the previous studies measurements of vertebral body morphology to train AI models, such as the ratio of the posterior height to the anterior height of vertebral bodies [18–22]. This approach is labor-intensive and time-consuming and results in a limited number of lateral cephalograms for training, while the sample size for training dramatically affects the final performance of the AI model. On the other hand, CVM is commonly evaluated in clinical settings by direct vision, allowing easy access to many samples for training. Currently, deep learning algorithms are the dominant technology in the field of artificial intelligence. It can perform feature extraction in an automated manner, which allows researchers to extract differentiated features with minimal domain knowledge and human effort. Therefore, combining the two makes it possible to train highly performing-AI models. In this study, experts created a dataset with a sample size of 10,200 and carefully assessed and collated it, resulting in a large and high-quality dataset.

In addition, unbalanced sample distribution (the difference in sample size between different classifications is more than ten times) will result in classifications with small sample sizes containing too few features, and extracting features from them will not be easy. Even if a classification model is obtained, it is prone to the problem of over-fitting due to over-reliance on limited data samples, and the robustness and accuracy of the model will be poor when the model is applied to new data. In this study, the sample size for each CVS classification in this dataset was 1700, effectively avoiding the problems associated with unbalanced samples.

In this study, we developed a new system, called the psc-CVM assessment system, for CVM assessment based on deep learning. Compared to the methods reported in the literature, this study assessed CVM directly according to extracted cervical vertebrae shape. The proposed system was designed as three parts with different roles, each operating in a specific order. 1) Position Network for locating the position of the second (C2), third (C3), and fourth (C4) cervical vertebrae; 2) Shape Recognition Network for recognizing and extracting the shapes of C2, C3, and C4; and 3) CVM Assessment Network for assessing CVM according to the shapes of C2, C3, and C4.

In the Position Network, YOLOv3 [24] was selected as the core of operation for this phase. Unlike R-CNN [29], Fast-R-CNN [30], and Faster-R-CNN [31], the latter is a one-stage target detection network. Compared with other mainstream target detection methods, YOLOv3 can achieve state-of-art accuracy and has a significant advantage in terms of speed.

In the Shape Recognition Network, this study proposed an EfficientNet-B0-based dense key point extraction network as the core of operation for this phase, using the link between contour key points and morphology to extract vertebral contours by predicting dense key points accurately. It must emphasize that spikes, or islands of bone, observed along the inferior border of the cervical bodies (C2, C3, and C4) in the anterior and posterior regions will interfere with the CVM assessment [25]. These small osseous structures are not part of the vertebral body but are free-floating. Spikes are often mistaken for a part of cervical bodies by young clinicians when they stage the cervical vertebrae. Using the contour of the vertebral body marked by senior experts as a training sample can make the system avoid such mistakes.

Due to the continuity of the skeletal growth process, there are transitional phases in adjacent growth cycles, such as the CVS3 phase containing growth spikes, and vertebral morphology often does not show typical morphology due to the active growth pattern. CVS uncertainty is unavoidable for samples close to the boundary of two stages [32]. This feature leads to inconsistent labeling results across clinicians and further affects the accuracy of the system. Setting the commonly used hard-label as the prediction classification target of the network could not be conducive to the network learning the features of the data better. Therefore, in the CVM Assessment Network, we use soft-label as the prediction target of the network [27]. Intuitively, this approach makes the system not wholly trust the label, so there are some ambiguous samples on edge in the data, and the model will not be affected by subjective classification, which is also why soft-label can increase the generalization of the system. At the same time, this method is also helpful in improving the accuracy of CVM assessment for the system.

Although this study tried several methods to improve the accuracy of CVM assessment of the system, the assessment effect of some CVS was still not ideal, resulting in an overall accuracy of 70.42%.

The results show that the F1 score of CVS2(0.6003) and CVS3(0.6340) is the lowest. The precise identification of these two CVSs has significant importance in clinical practice. In the subsequent research, we will improve the algorithm for this issue and include factors such as intervertebral disc space and dental age to enhance the system's accuracy in identifying CVS2 and CVS3.

And the whole system is not end-to-end but divided into three steps. This working framework would carry errors generated in the previous step into the next. Moreover, we found that the overall ICC between the psc-CVM assessment system and the expert panel was 0.946, indicating that the system in this study was significantly consistent with the expert panel in the CVM assessment.

In this study, the system only focused on the vertebrae. At the same time, other regions in the lateral cephalograms may have valid information to help CVM assessment, which was not included in training in this study. In subsequent studies, it may be possible to unify the entire process into an end-to-end all-in-one system, coordinate and optimize the various steps, add valid information related to CVM, and improve the system's accuracy.

The above results indicate that the psc-CVM assessment system in this study is stable and significantly consistent with the expert assessment results. Nevertheless, in a clinical setting, where diagnosis and treatment planning require the integration of various factors, the system still needs to be able to make systematic decisions like an expert due to the limitations of the technology. Therefore, the psc-CVM assessment system is only used as an auxiliary guidance tool in the clinical setting, providing valuable reference information for clinicians who lack the clinical experience. The system will make the treatment process more precise and effective and is now available for integration into the software of medical companies for free use by clinicians. In addition, the system will be regularly monitored and upgraded in future studies to ensure its stability in real-world applications.

## Conclusion

This study establishes a fully automated, high-accuracy CVM assessment system called the psc-CVM assessment system based on deep learning. The results showed that the system achieved high accuracy in CVM assessment. The system in this present study was significantly consistent with expert panels in CVM assessment, indicating that the system can be used as an efficient, accurate, and stable diagnostic aid to provide a clinical aid for determining growth and developmental stages by CVM.

### Supplementary Information

**Additional file 1.** 

## Data Availability

The datasets generated and/or analysed during the current study are not publicly available due to patient privacy and data security but are available from the corresponding author on reasonable request.

## Abbreviations

CVM: Cervical vertebral maturation
AUC: The area under the curve
AI: Artificial intelligence
CVS: Cervical vertebral maturation stage
P-R curve: Precision-recall curve
ROC curve: Receiver operating characteristic curve
ICCs: Intragroup correlation coefficients
