# Supplementary material for: The psc-CVM assessment system: A three-stage type system for CVM assessment based on deep learning
Source: BMC Oral Health. 2023 Aug 12;23:557. doi: 10.1186/s12903-023-03266-7 (PMC10422791; doi:10.1186/s12903-023-03266-7)
Supplement: Supplementary file 1 — Additional file 1. [file 12903_2023_3266_MOESM1_ESM.docx]

**The psc-CVM assessment system: A three-stage type system for CVM assessment based on deep learning**

***Training Details of the psc-CVM assessment system***

***Position Network***

First, YOLOv3[1] was selected as the target detection network to identify and label the locations of all vertebrae appearing in the input. Unlike R-CNN[2], Fast-R-CNN[3], and Faster-R-CNN[4], which generate the possible bounding boxes of the object to be detected through candidate regions or sliding windows and then use classifiers to determine the confidence and class of the object, the YOLO series of networks treats the detection task as a regression problem. It can predict information such as object frame coordinates, class of contained objects, and confidence level directly from the image. It is an end-to-end object detection method.

Specifically, the backbone network extracted the input image to obtain feature image 1 after convolution and feature image 2 after a 1×1 convolution and upsampling operation. Furthermore, feature image 3 was obtained by stitching with the same scale of features in the backbone. The number of channels of the feature image output is $B\times\left( 4+1+C \right)$, where $B$ denotes the number of anchors at each grid point, and 4, 1, and C correspond to the 4-dimensional prediction frame values$t_{x}, t_{y},t_{w},t_{h}$, the 1-dimensional confidence level, and the C-dimensional target category, respectively. In the experiment, the vertebrae were divided into C2 and non-C2 vertebrae by morphology (the number of categories was two); the dimensions of the three feature images were 1/32, 1/16, and 1/8 of the original images.

After obtaining the three feature images, the shape and position parameters of the object detection boxes can be decoded by combining the anchor.

$$\begin{aligned} b_{x}=\sigma\left( t_{x} \right)+c_{x} , b_{y}= \sigma\left( t_{y} \right)+c_{y} \#\left( 1 \right) \end{aligned}$$

$$\begin{aligned} b_{w}=p_{w}e^{t_{w}} , b_{h}=p_{h}e^{t_{h}} \#\left( 2 \right) \end{aligned}$$

where $c_{x}, c_{y}$ are the coordinates of the upper left corner of the anchor, $\sigma\left( t_{x} \right), \sigma(t_{y})$ are the offset of the center of the rectangular box relative to the upper left corner of the grid, $\sigma$ is the sigmoid activation function, and $p_{w}, p_{h}$ are the width and height of the anchor, respectively.

For a 256×256-dimensional input, a total of 7111 frames with their prediction categories and confidence levels will be decoded. In the training phase, for each truth-valued object, the prediction frame with the highest IOU value was marked as the positive example of the object to calculate the confidence, detection box, and losses of the classes. For the rest of the predictions, only the loss with a confidence label of 0 was calculated for prediction frames with IOU below the threshold, and non-positive case predictions with IOUs above the threshold were not added to the calculation of loss. In the inference stage, after decoding the prediction, a threshold was set to filter the detection boxes with low confidence. Then, the cervical vertebrae detection results were obtained by NMS (non-maximum suppression). Based on the predicted position of C2, multiple detection boxes can be identified as C3, C4, C5, and C6. from the center to the edge of the image in order. The threshold value was set to 0.5.

The objective function in training is the sum of the losses of the three feature images. For each feature image, the objective function consists of coordinate error, confidence error, and classification error, and we adjust the ratio of the three by $\lambda_{coor}, \lambda_{cls}$.

$$\begin{aligned} \mathcal{L=}\lambda_{coor} \mathcal{L}_{coor}+\mathcal{L}_{obj}+\lambda_{cls} \mathcal{L}_{cls}\#\left( 3 \right) \end{aligned}$$

The coordinate error includes the center and width and height, which are calculated by MSE. The confidence error takes into account both object and no-object components. The no-object confidence loss has a weighting factor $\lambda_{noobj}$, which is used to balance the phenomenon that the area not containing the object is detected much larger than the area containing the object and to prevent the network from predicting frames that do not contain objects. Confidence and classification prediction are dichotomous tasks, so cross entropy is used as the loss function.

$$\begin{aligned} \mathcal{L}_{corr}=\sum_{i}^{S^{2}} \sum_{k}^{B} \mathbb{l}_{ij}^{obj}\left[ \left( t_{x}-\hat{t}_{x} \right)^{2}+\left( t_{y}-\hat{t}_{y} \right)^{2} \right]+\mathbb{l}_{ij}^{obj}\left[ \left( t_{w}-\hat{t}_{w} \right)^{2}+\left( t_{h}-\hat{t}_{h} \right)^{2} \right]\#\left( 4 \right) \end{aligned}$$

$$\begin{aligned} \mathcal{L}_{obj}=-\sum_{i}^{S^{2}} \sum_{k}^{B} \mathbb{l}_{ij}^{obj}\log c_{ij}+\lambda_{noobj}\mathbb{l}_{ij}^{noobj}\log\left( 1-c_{ij} \right)\#\left( 5 \right) \end{aligned}$$

$$\begin{aligned} \mathcal{L}_{cls}=-\mathbb{l}_{ij}^{obj}\sum_{c\in classes} p_{ij}^{'}\left( c \right)\log p_{ij}\left( c \right)+\left( 1-p_{ij}^{'}\left( c \right) \right)\log\left( 1-p_{ij}\left( c \right) \right)\#\left( 6 \right) \end{aligned}$$

***Shape Recognition Network***

The morphology of the vertebrae is the focus of clinicians' attention in the diagnostic process. Therefore, this paper proposes the accurate extraction of vertebral contours by predicting dense key points using the relation between contour key points and morphology, modeling the morphology extraction as a heatmap regression problem.

Extract 1000 vertebrae images in the previous step, and let the expert panel sketch their contours. The data obtained is used for the network training at this stage. The vertebral contours and their significant corner points were marked on the X-ray images by the clinicians. The outlines were divided into multiple curves according to the corner points, and each curve was fitted with a separate spline. The fitted smooth splines were sampled at equal intervals according to the accuracy requirements to obtain multiple key point markers. We used lp, la, up, and ua to denote the four corner points (Appendix Table 1). Due to the blurred imaging of the superior region of the C2 vertebra, the actual contours were difficult to distinguish, so only the anterior and posterior edges of the vertebral body parts and the complete inferior edge were considered. The sampling results are shown in Appendix Figure 1.

EfficientNet[5] generates the baseline model B0 using MnasNet[6] implemented by a reinforcement learning algorithm and then uses a compound scaling method to simultaneously adjust the three network configuration parameters of model depth, width, and image resolution to search for architecture with appropriate accuracy under preset memory and computation constraints.

An encoder is constructed based on the first three downsampling and convoluting processes to extract the multiscale features of the image, followed by feature fusion to obtain the prediction at the same scale as the original image. The network learns to regress N heatmaps $h_{i}$ by minimizing the mean square error between the predicted and real heatmaps.

We used the true coordinates $\dot{x_{i}}$ of landmarks $L_{i}, i=\left\{ 1, \ldots, N \right\}$ to generate a heatmap as the target of the network regression, and the heatmap generated with the Gaussian map function can be expressed as follows:

$$\begin{aligned} g\left( x; \sigma_{i} \right)=\frac{\gamma}{\left( 2\pi\right)^{\frac{d}{2}}\sigma_{i}^{d}}\exp\left( -\frac{\left\| x-\dot{x_{i}} \right\|_{2}^{2}}{2\sigma_{i}^{2}} \right) \#\left( 7 \right) \end{aligned}$$

where γ is used to avoid instability due to minimal values of the Gaussian function during the training process.

Three feature images of 1/2, 1/4, and 1/8 steps were taken from the encoder and concatenated with 1, 2, and 4 times upsampling, respectively, to obtain an output of depth N after 1×1 convolution and sigmoid activation.

As in the previous step, the network was divided into two settings according to the vertebrae morphology. For C2, a total of 2 keypoints were identified at the posterior edge, lower edge, and anterior edge, and for the other vertebrae images, 40 keypoints were detected uniformly at the upper, lower, posterior and anterior edges.

The base block of EfficientNet, MBCon, consists of 1×1 convolution for dimensionality up, k×k (3 in this experiment) Depthwise convolution, SE module (Squeeze-and-Excitation Module), 1×1 convolution for dimensionality down, and Dropout layer in order, with the addition of a shortcut from input to output. All convolutions contain BN, except for the last convolution in the block, and all convolution knot operations have Swish activation.

The squeeze operation in the SE module contains only global average pooling. The excitation operation consists of two fully connected layers and swish activation. The first fully connected layer has 1/4 of the number of input channels of the MBConv block and is followed by the swish activation function; the second fully connected layer has the same number of nodes as the output channels of the depthwise convolution layer and uses the sigmoid activation function. The result was multiplied by the input feature image as the output.

***CVM assessment Network***

We combined the results of the first two steps. The images in the C2-C4 range were cropped according to the prediction rectangle in the first step. The corresponding vertebral binary masks were generated based on the output of the second step, which was concatenated and used as inputs.

This phase builds the classification model, using ConvNext[7] as the backbone. Due to the continuity of the skeletal growth process, there are transitional stages of adjacent growth cycles. For example, some samples may be in the transition between CVS3 and CVS4. Therefore， CVS uncertainty is unavoidable for samples close to the boundary of two stages. If the prediction classification target of the network is set to the commonly used hard-label, it is not conducive to the network learning the features of the data better, so we use soft-label as the prediction target of the network. Extending the classification head to 8 dimensions, i.e., a 1x1x8 output tensor, the objective of the network is set to minimize the CrossEntropy Loss to the predicted target.

**Appendix Table 1** The cervical vertebrae reference points.

| Point name | Description |
| --- | --- |
| lp | The posterior point of the lower edge of the vertebra |
| la | The anterior point of the lower edge of the vertebra |
| up | The posterior point of the upper edge of the vertebra |
| ua | The anterior point of the upper edge of the vertebra |


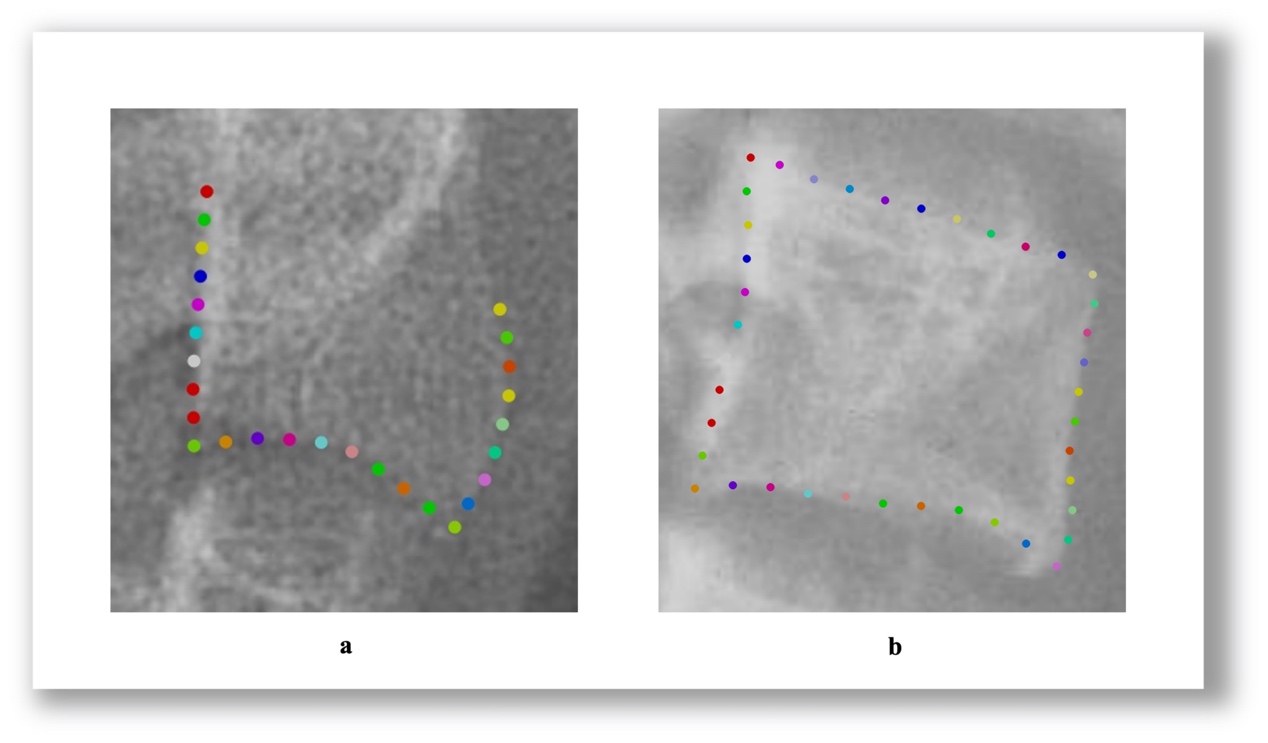


**Appendix Figure 1.** A magniﬁed view of the anatomic landmarks used for cervical vertebral feature extraction using labeling software. a) shows the results of sampling on C2 vertebrae. b) shows the results of sampling on C3 or C4 vertebrae.

**Reference**

1. Redmon J, Farhadi A: **YOLOv3: An Incremental Improvement**. 2018.

2. Girshick R, Donahue J, Darrell T, Malik J: **Region-Based Convolutional Networks for Accurate Object Detection and Segmentation**. *TPAMI* 2016, **38**(1):142-158.

3. Girshick R: **Fast R-CNN**. In*.*: IEEE; 2015: 1440-1448.

4. Ren S, He K, Girshick R, Sun J: **Faster R-CNN: Towards Real-Time Object Detection with Region Proposal Networks**. *TPAMI* 2017, **39**(6):1137-1149.

5. Tan M, Le QV: **EfficientNet: Rethinking Model Scaling for Convolutional Neural Networks**. 2019.

6. Tan M, Chen B, Pang R, Vasudevan V, Sandler M, Howard A, Le QV: **MnasNet: Platform-Aware Neural Architecture Search for Mobile**. 2018.

7. Liu Z, Mao H, Wu CY, Feichtenhofer C, Darrell T, Xie S: **A ConvNet for the 2020s**. *arXiv e-prints* 2022.
